# Analysis of PERV-C superinfection resistance using HA-tagged viruses

**DOI:** 10.1186/s12977-023-00630-x

**Published:** 2023-08-21

**Authors:** Merle Flecks, Nicole Fischer, Jacomina Krijnse Locker, Ralf R. Tönjes, Antonia W. Godehardt

**Affiliations:** 1 Division of Haematology, Cell and Gene Therapy, Paul-Ehrlich-Institut, Langen, Germany; 2 Loewe-DRUID Research Group, Electron Microscopy of Pathogens, Paul-Ehrlich-Institut, Langen, Germany

**Keywords:** Porcine endogenous retrovirus, Superinfection resistance, Xenotransplantation, Virological safety, Env protein, HA-tag

## Abstract

**Background:**

Using pigs as organ donors has advanced xenotransplantation to the point that it is almost ready for clinical use. However, there is still a zoonotic risk associated with xenotransplantation, and the potential transmission of porcine endogenous retroviruses needs to be surveyed. Despite significant attempts to eliminate this risk, by the selection of PERV-C free pigs with low expression of PERV-A, -B, and by the genome-wide inactivation of PERV using CRISPR/Cas9, the impact of superinfection resistance (SIR) was not investigated. SIR is a viral trait that prevents reinfection (superinfection). For PERV, the underlying mechanism is unclear, whether and how cells, that harbor functional PERV, are protected. Using PERV-C(5683) as a reference virus, we investigated SIR in a newly developed in vitro model to pursue the mechanism and confirm its protective effect.

**Results:**

We developed three PERV-C constructs on the basis of PERV-C(5683), each of which carries a hemagglutinin tag (HA-tag) at a different position of the envelope gene (SP-HA, HA-VRA, and RPep-HA), to distinguish between primary infection and superinfection. The newly generated PERV-C(5683)-HA viruses were characterized while quantifying the viral RNA, reverse transcriptase activity, protein expression analysis, and infection studies. It was demonstrated that SP-HA and RPep-HA were comparable to PERV-C(5683), whereas HA-VRA was not replication competent. SP-HA and RPep-HA were chosen to challenge PERV-C(5683)-positive ST-IOWA cells demonstrating that PERV-C-HA viruses are not able to superinfect those cells. They do not integrate into the genome and are not expressed.

**Conclusions:**

The mechanism of SIR applies to PERV-C. The production of PERV-C particles serves as a defense mechanism from superinfection with exogenous PERV-C. It was demonstrated by newly generated PERV-C(5683)-HA clones that might be used as a cutting-edge tool. The HA-tagging of PERV-C is novel, providing a blueprint for the tagging of other human tropic PERV viruses. The tagged viruses are suitable for additional in vitro and in vivo infection studies and will contribute, to basic research on viral invasion and pathogenesis. It will maintain the virus safety of XTx.

**Supplementary Information:**

The online version contains supplementary material available at 10.1186/s12977-023-00630-x.

## Background

Pig-to-human xenotransplantation (XTx) is a promising strategy to overcome the limited availability of human allografts [1–3]. A human patient had the first genetically modified pig heart transplant in 2022 [4]. Unfortunately, the finding of porcine cytomegalovirus (PCMV) in the patients’ blood confirmed that viral transmission is difficult to prevent [4, 5]. While designated pathogen free (DPF) breeding can apparently eliminate most of the porcine viruses, porcine endogenous retroviruses (PERV) can not be excluded. They are stably integrated in the pig genome [6–9]. While polytropic PERV-A and -B can infect human cells in vitro, infection with ecotropic PERV-C is restricted to porcine cells [6–13]. PERV-C raises concerns as it may recombine with PERV-A to a highly infectious, human tropic PERV-A/C variant that replicates at high titers [14–17]. Due to the evolutionary age most of the proviral PERV loci that have been identified in the porcine genome are defective [7, 18], and the number of complete, full length sequences that were functionally characterized is comparably small [19–26]. The hypothesis if dysfunctional proviruses may reassemble was considered but not confirmed. So far no functional particles have been observed in cells that are free of functional provirus [7, 27]. It underlines, that the absence of functional PERV is obligatory to guarantee safe XTx [28–33]. With the genome‐wide inactivation of PERV using CRISPR/Cas9 in 2015 [34, 35] and the finding that the inactivated cell line (PK15 *clone 15*) was resistant against PERV-A, -B, -A/C superinfection (SI), superinfection resistance (SIR) was discussed as a protection mechanism [36].

Generally, retroviruses can create SIR in cells by establishing an interference mechanism after the initial (primary) infection that prevents infected cells from being superinfected by a similar or same type of virus [37]. SIR is likely to result from receptors being hidden or downregulated after interaction with the known virus’s glycoprotein, either intracellularly or at the cellular surface [37–40]. Retroviruses like murine leukemia virus (MuLV), human immunodeficiency virus (HIV) and foamy virus (FV) use Env-mediated SIR mechanisms, so a similar mechanism may be assumed for PERV [36, 37]. It was shown that proviral gene products can mediate the restriction of viral replication [37]. In consequence, targeted expression of virus specific genes should be considered as a potential protection mechanism against retroviral infections. Several examples have been published.

In 1975, Suzuki discovered the *Fv4* gene, which encodes a retrovirus-related Env polyprotein from the *Fv4* locus containing the mid *pol*-region, *env* and the 3′-long terminal repeat (LTR) [41–43]. In vitro data of NIH 3T3 cell lines stably transfected with the cloned *Fv-4 env* gene confirmed SIR to ecotropic MuLVs [44]. In vivo studies using transgenic mice carrying the *Fv4* gene affirmed this result. The resistance correlated with the *Fv-4 env* expression level. It was assumed that the MuLV receptor is probably masked by the Fv4-Env complex [45].

For PERV, an evidence for PERV SIR mechanism was shown for reverse transcriptase (RT) defective PERV-A and -B released from a porcine kidney cell line (PK15 *clone 15* cells) that had been inactivated by CRISPR/Cas9 [34, 36]. These cells still produce complete but dysfunctional virus particles, which were irregularly shaped and apparently immature [36].

The cells could not be reinfected and remained RT negative. However, the question if foreign PERV-A, -B, -C or -A/C was able to enter the cells or not was not sufficiently answered since all viruses used for this analysis were untagged wildtype viruses and nearly indistinguishable from the receding viruses.

In this study, we focused on PERV-C(5683), as a reference virus. We generated, for the first time, HA-tagged PERV-C viruses, alongside providing a blueprint for PERV tagging. Viruses have been tested for functionality and SI in an in vitro assay on primary infected and non-infected porcine ST-IOWA cells. The results demonstrate that ST-IOWA cells primarily infected with functional PERV-C(5683) subsequently show resistance to infection with the same type of virus. The resistance probably will be conveyed during the early phase of infection, as there was no integration to the host genome. Our data prove that SIR also exists for PERV-C and that SIR is mediated by functional as well as dysfunctional PERV particles. A finding that opens up new strategies while using viral components for cellular resistance [34–36, 46–48]. In addition, the tagged virus is a novel tool and data are suitable for the generation of e.g. PERV-A, -B and -A/C clones to further analyze PERV infection mechanism, perhaps in vivo*.*

## Results

### Generation and functional analysis of HA-tagged PERV-C(5683)

**Fig. 1:**
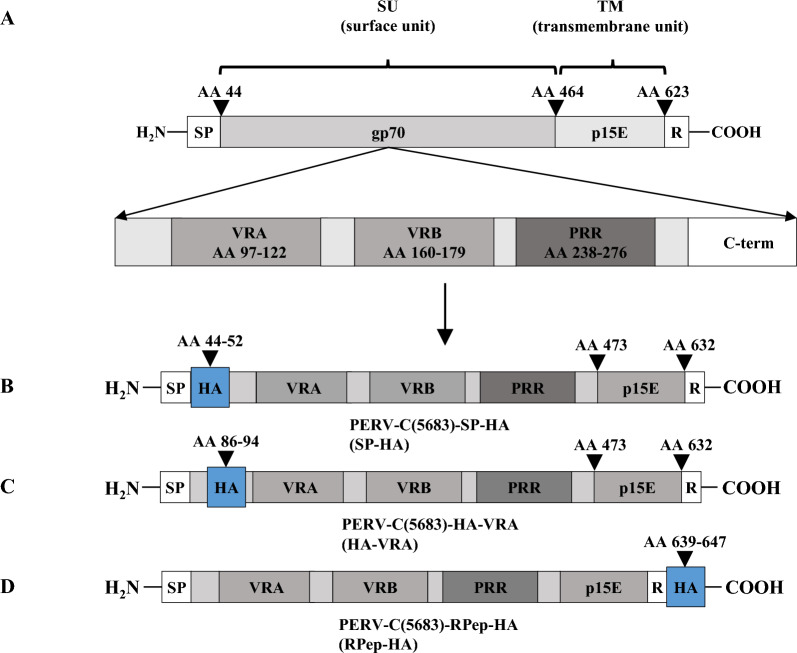
Structure and design of PERV-C(5683)-HA constructs. The HA-tag was inserted into the *env* gene of PERV-C(5683) (KY352351.2) by site directed mutagenesis. **A** Regular structure of PERV-C(5683) *env*. PERV-C(5683) molecular clones carrying the HA-tag **B** C-terminal to the SP at amino acid (AA) 44–52 (SP-HA), **C** N-terminal to the VRA at AA 86–94 (HA-VRA) or **D** C-terminal to the RPep at AA 639–647 (RPep-HA). The integration sites of HA were chosen on the basis of functional studies [9, 25, 56, 57] and secondary structure analyses using the open source tool PSIPRED [55]

**Fig. 2:**
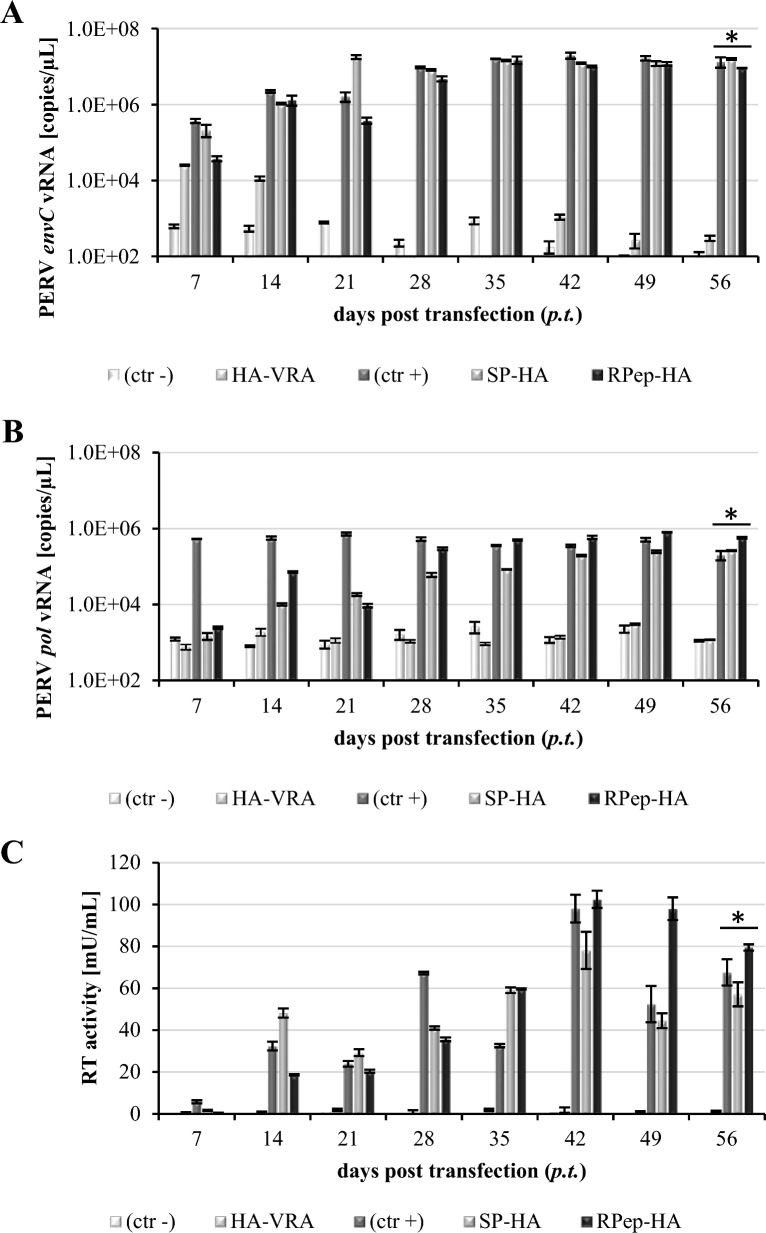
Quantification of vRNA and RT activity in ST-IOWA cells transfected with HA-tagged PERV-C(5683). Cells were monitored for 56 days by collecting cell culture SNs weekly. Expression of PERV-C(5683) **A** *env* and **B** *pol* vRNA for SP-HA and RPep-HA showed equal levels compared to the positive control PERV-C(5683) (ctr+). HA-VRA transfected cells revealed only basal expression comparable to the PERV-C negative ST-IOWA cells (ctr−). **C** Consistent to the presence of vRNA PERV-C(5683), SP-HA and RPep-HA became RT positive within two weeks after transfection. HA-VRA remained negative for RT activity. Significant means of < 0.05 according to Welch’s t-test are indicated by (*). Analysis were performed in triplicates

By the insertion of an HA-sequence (YPYDVPDYA) in the *env* gene of the PERV-C(5683) backbone (KY352351.2) [26] PERV-C(5683)-HA viruses were generated. The HA-tag was positioned (i) downstream of the signal peptide (SP) yielding PERV-C(5683)-SP-HA (SP-HA), (ii) upstream of the variable region A (VRA) generating PERV-C(5683)-HA-VRA (HA-VRA) and (iii) downstream of the R-peptide (RPep) yielding PERV-C(5683)-RPep-HA (RPep-HA) (Fig. 1, Table 1).The three HA-tagged PERV-C(5683) constructs, namely SP-HA, HA-VRA and RPep-HA, were tested for functionality by transfection of ST-IOWA cells and subsequent analysis of (i) viral expression, (ii) RT activity, (iii) stable integration of the tagged provirus into the host genome, (iv) morphology of viral particles as well as (v) infectious capacity. SP-HA and RPep-HA showed expression of PERV-C *env* and *pol* viral RNA (vRNA) 14 days post transfection (*p.t.*) (Fig. 2A, B). Levels of vRNA were comparable to those of PERV-C(5683), with approximately 10^6^–10^7^ copies/µL (*envC*) and 10^5^–10^6^ copies/µL (*pol*) 56 days *p.t.*. HA-VRA revealed a low expression for *envC* and *pol* of approximately 10^2^ and 10^3^ copies/µL at 56 days *p.t.*, that is comparable to the basal expression measured for native ST-IOWA cells [9]. RT activity was detectable 14 days *p.t.* (Fig. 2C). RT activities of PERV-C(5683), SP-HA as well as RPep-HA stayed on comparable levels in the time of quantification reaching activities of 57 mU/mL for SP-HA, 79 mU/mL for RPep-HA and 67 mU/mL for PERV-C(5683) 56 days *p.t.*. Maximal RT activities were reached at day 42 with 78 mU/mL for SP-HA, 102 mU/mL for RPep-HA and 98 mU/mL for PERV-C(5683). HA-VRA showed no RT activity. Native ST-IOWA cells served as negative control (ctr−).

**Table 1:** Oligonucleotide primers used for cloning of PERV-C(5683)-HA plasmids

Primer	Target gene	Sequence 5′-3′*	Accession No	Nucleotide pos
Env_SP-HA_F	PERV *envC*	**GCCGGATTATGCG**ATGCGCATA GGAGACAGCCTG	KY352351.2	6507–6524
cEnv_SP-HA_R	PERV *envC*	**ACATCATACGGATA**ACCATTAG TCTGAGAGGTTATTGACAGAG	KY352351.2	6484–6506
Env_HA-VRA_F	PERV *envC*	**GCCGGATTATGCG**GATCTATACG TTTGCCTCAGATCAGTTATTCC	KY352351.2	6633–6664
cEnv_HA-VRA_R	PERV *envC*	**ACATCATACGGATA**AGGCCACC AGGTTCCTAAAGG	KY352351.2	6612–6632
Env_RPep-HA_F	PERV *envC*	**GCCGGATTATGCG**TAGCTCTACC AGTTCTAAGATTAGAAC	KY352351.2	8292–8318
cEnv_RPep-HA_R	PERV *envC*	**ACATCATACGGATA**GCGGCCAGC TTCCCTGCT	KY352351.2	8274–8291

^*^Nucleotides of the HA-tag, introduced by site directed mutagenesis are shown in bold

The results show that the insertion of an HA-tag close to the signal peptide (SP-HA) or to the membrane anchor (RPep-HA) had no effect on virus functionality. An introduction N-terminal to the VRA region restrained viral replication. Due to the lack of vRNA expression, HA-VRA was examined for its ability to integrate into the host genome as a provirus. To this end, genomic DNA (gDNA) from ST-IOWA cells transfected with HA-VRA (21 days *p.t.*) was analyzed for HA-VRA integration by PCR using *envC*-specific primers. No integration of HA-VRA provirus into the host genome was detected (data not shown). Therefore, HA-VRA was excluded from further analysis.

**Fig. 3:**
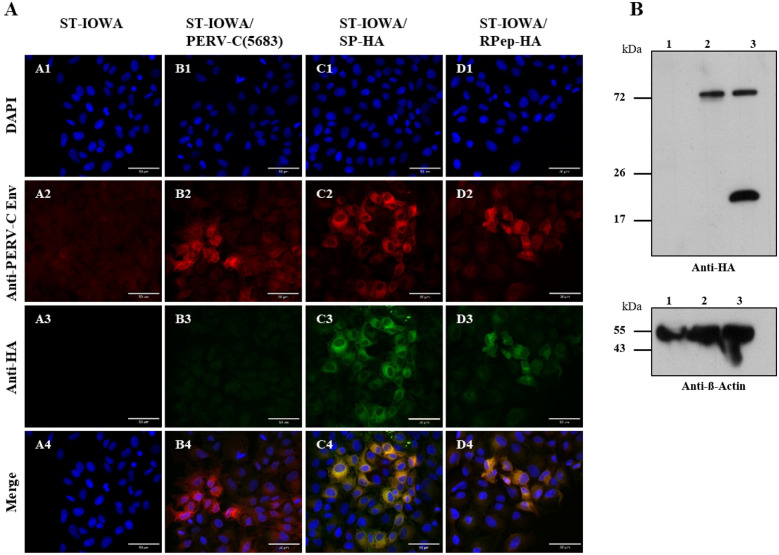
Immunofluorescence and WB analysis of ST-IOWA cells transfected with PERV-C. **A** Immunofluorescence of SP-HA and RPep-HA transfected ST-IOWA cells using anti-HA-tag and anti-EnvC directed antibodies, 56 days *p.t.*. A1-4: ST-IOWA cells (ctr−), B1-4: PERV-C(5683) positive ST-IOWA cells (ctr+), C1-4: SP-HA and D1-4: RPep-HA transfected ST-IOWA cells. PERV-C Env proteins (red fluorescence) were detected for all cells except for the non-transfected control ST-IOWA. PERV-C Env-HA proteins were only observed for cells transfected with SP-HA or RPep-HA, as indicated by green fluorescence. **B** Detection of HA proteins by WB analysis in cell lysates of ST-IOWA cells transfected with SP-HA or RPep-HA harvested at day 56 *p.t*. (lane 1: ST-IOWA (ctr−), lane 2: SP-HA and lane 3: RPep-HA). Anti‐beta‐actin was used as loading control. PERV-C Env-HA precursor is expected at 72.1 kDa, its cleaved products SP-HA-SU at 52.3 kDa and TM-HA at 20.9 kDa. PERV-C Env-HA precursor proteins were detected for both, SP-HA and RPep-HA. SP-HA-SU shows only slight size variation compared to the precursor protein. For RPep-HA, both the precursor and the cleaved product were detected

Recombinant SP-HA and RPep-HA were analyzed for stable HA-tag integration and protein expression by immunofluorescence (IF) experiments and western blot (WB) analysis 56 days *p.t*. (Fig. 3). IF showed the presence of EnvC-HA proteins in transfected cell lines ST-IOWA/SP-HA and ST-IOWA/RPep-HA (Fig. 3A).

WB analysis using an anti-HA-tag antibody confirmed that EnvC-HA is present as a precursor and in its processed forms namely the surface unit (SU) and the transmembrane unit (TM) (Fig. 3B). Depending on the HA-tag position, the detection of the cleaved variant is either N-terminal restricted to SU or C-terminal to TM. As expected, SP-HA (lane 2) showed one large protein band at approximately 70 kDa representing the precursor EnvC-HA (72.1 kDa). The precursor and the processed form SP-HA-SU (52.3 kDa) could not be differentiated. For RPep-HA, the precursor EnvC-HA (72.1 kDa) as well as TM-HA (20.9 kDa) were detected.

**Fig. 4:**
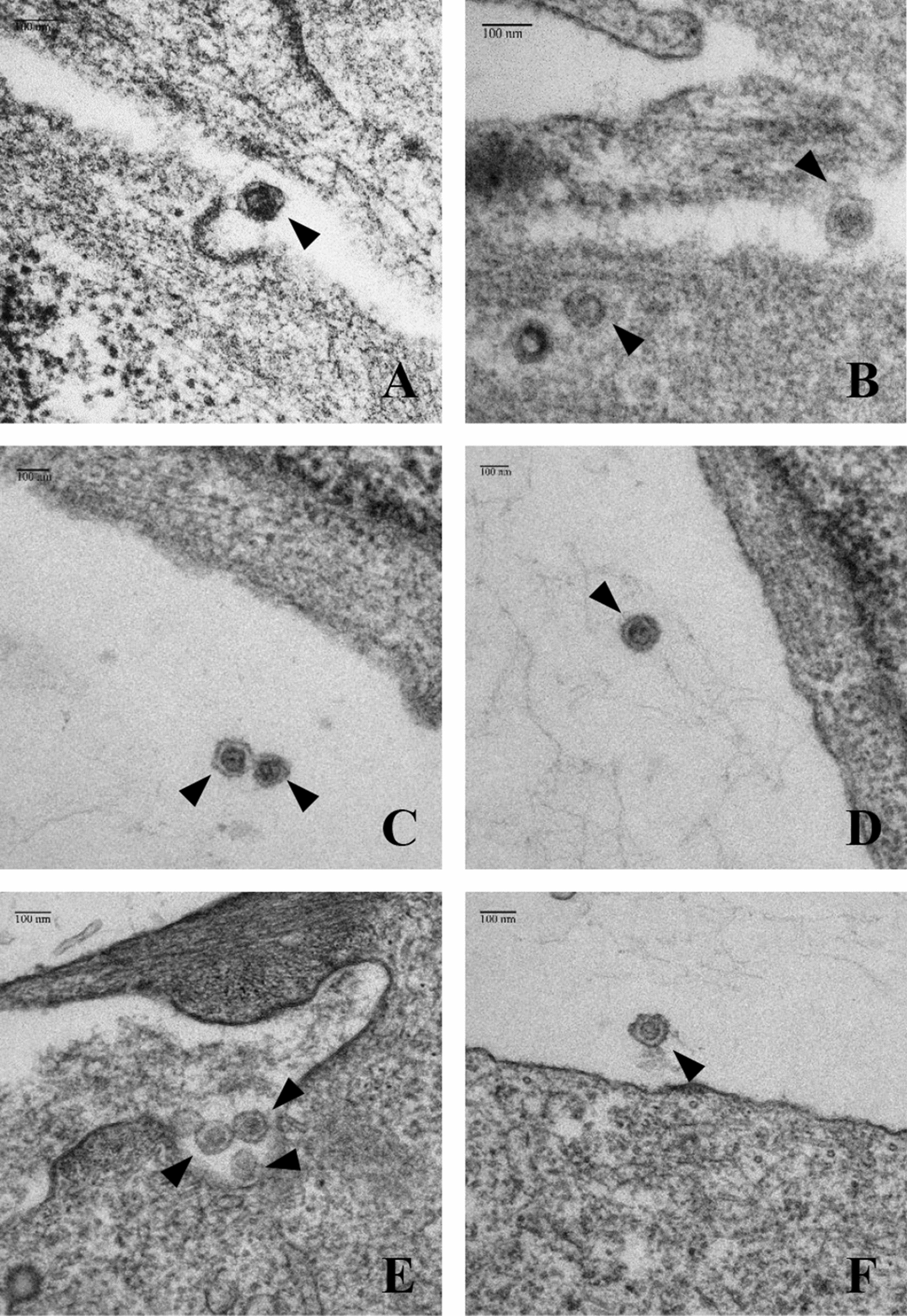
Electron microscopy of PERV-C virus particles in transfected ST-IOWA cells. **A**, **B** PERV-C(5683) particles, **C**, **D** virus in ST-IOWA cells transfected with SP-HA, **E**, **F** viral particles in ST-IOWA cells transfected with RPep-HA. Virions are indicated by black arrow heads. All viral particles show an identical morphology with a dense, collapsed core surrounded by evenly rounded capsids

Electron microscopy (EM) was used to demonstrate that the integration of an HA-tag did not change the morphology of the virus particles (Fig. 4). EM of SP-HA and RPep-HA revealed a characteristic morphology of viral particles, showing a dense, collapsed core surrounded by evenly rounded capsid that is comparable to native PERV-C(5683). EM of ST-IOWA cells did not show any virus or virus-like particles (data not shown).

**Fig. 5:**
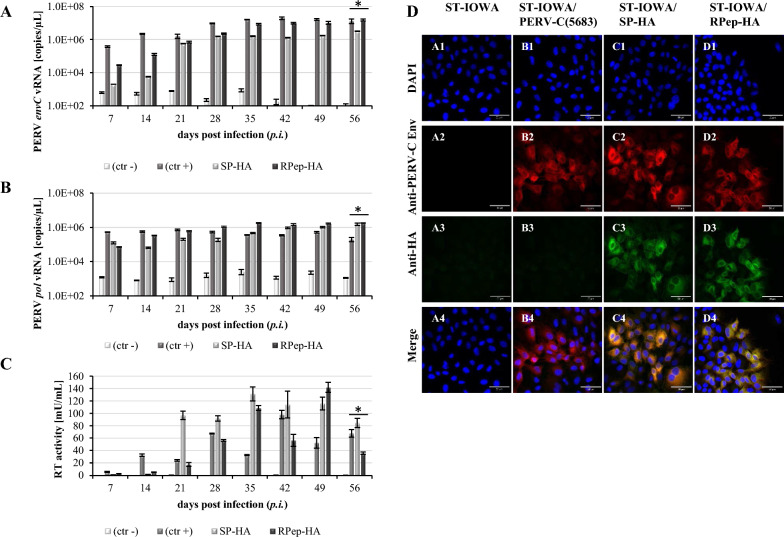
Infection of ST-IOWA cells with cell culture SN of HA-tagged PERV-C(5683). Cells were incubated for 24 h with SN and infection was monitored for 56 days on a weekly basis. Quantification of **A** *envC* and **B** *pol* vRNA showing increasing copy numbers from the first week on for PERV-C(5683) that was used as positive control as well as for SP-HA and RPep-HA. Non-infected ST-IOWA cells stayed on levels of basal expression. **C** Quantification of RT activity. RT activity could be detected from day 14 *p.i.* for all cell lines except for non-infected ST-IOWA cells that served as negative control (ctr−). **D** Immunohistochemistry of PERV-C-HA infected ST-IOWA cells at day 56 *p.i.* using PERV-C Env and HA-tag directed antibodies. **A1**–**4** ST-IOWA (ctr−), **B1**–**4** PERV-C(5683) infected ST-IOWA cells (ctr+), **C1**–**4** SP-HA infected ST-IOWA cells, **D1**–**4**: RPep-HA infected ST-IOWA cells. In accordance with the transfection studies PERV-C Env-HA was only present in ST-IOWA cells infected with SP-HA or RPep-HA, indicated in green and yellow. Significant means of triplicates of < 0.05 according to Welch’s t-test are indicated by (*)

In continuation, the functionality of the PERV-C-HA viruses SP-HA and RPep-HA was demonstrated by incubation of native ST-IOWA cells with virus containing supernatant (SN) derived from the transfected cell lines (Table 2). Infection of ST-IOWA cells with SN from ST-IOWA/PERV-C(5683) cells served as positive control. The infection progress was monitored weekly, by quantification of PERV-C *env* and PERV *pol* vRNA expression, RT activity measurement and IF (Fig. 5). Irrespective of the PERV-C variant used for infection, vRNA expression was observed from the second week on reaching levels between 10^5^–10^6^ copies/µL (Fig. 5A, B) and RT activities between 15 and 50 mU/mL were detected from the third week on (Fig. 5C). In addition, infected and non-infected cells were distinguished by IF at day 56 days post infection (*p.i.*) using an anti-HA-tag antibody and anti-EnvC peptide directed antiserum (Fig. 5D).

**Table 2:** PERV-C producing cell lines used for the infection of ST-IOWA and superinfection of ST-IOWA/PERV-C(5683) cells

PERV-C	Producer cell line	RT activity ( mU/mL)	References
SP-HA (transfected cell line, 21 *p.t.*)	ST-IOWA/SP-HA	29.26	KY352351.2 (modified)
RPep-HA (transfected cell line, 28 *p.t.*)	ST-IOWA/RPep-HA	32.76	KY352351.2 (modified)
SP-HA (producer cell line)	ST-IOWA/SP-HA	59.19	KY352351.2 (modified)
RPep-HA (producer cell line)	ST-IOWA/RPep-HA	62.94	KY352351.2 (modified)

### AlphaFold2 based structural analysis of HA-tagged PERV-C

PERV-C(5683) Env as well as the three HA-tagged EnvC were structurally analyzed and compared using AlphaFold2 software [49] to gain insight into possible changes in the PERV-C Env structure that resulted in functional or non-functional viruses.

**Fig. 6:**
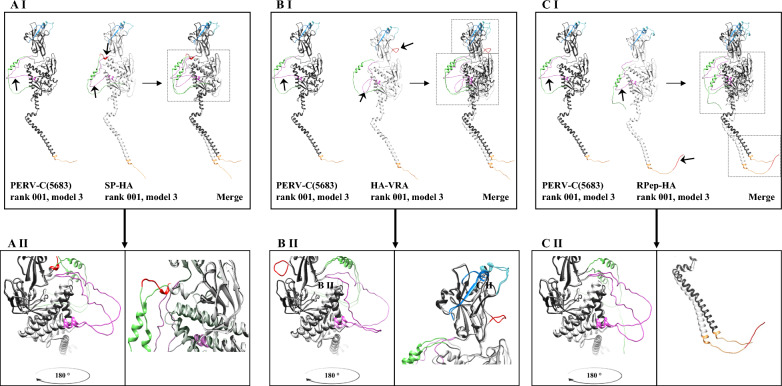
Structural prediction of native PERV-C(5683) (KY352351.2) and HA-tagged PERV-C(5683) variants. Structures were predicted using AlphaFold2 [46] and compared using a MUSCLE alignment. PERV-C(5683) is colored in dark gray and PERV-C(5683)-HA variants are shown in light gray. Major sequence motifs are color-coded SP (green), VRA (light blue), VRB (dark blue), PRR (purple), and RPep (yellow). The HA-tag is colored in red. **A** Comparison and alignment of PERV-C(5683) and SP-HA. **AI** Whole proteins. **AII** Detailed view of predicted structural changes. Insertion of the HA-tag in SP-HA resulted in a shift of the PRR (left) and the formation of an additional alpha helix adjacent to SP (right). **B** Comparison and alignment of PERV-C(5683) and HA-VRA. (B I), whole proteins. (B II), detailed view of predicted structural changes. A twist in the PRR was observed in HA-VRA, resulting in a compressed loop compared with PERV-C(5683) (left). In addition, an intrinsically twisted loop was formed by the HA-tag (right). **C** Comparison and alignment of PERV-C(5683) and RPep. **CI** Whole proteins. **CII** Detailed view of predicted structural changes. RPep-HA shows a slightly shifted structure compared with PERV-C(5683). The main differences are in the SP, PRR (left) and RPep (right) and are indicated by arrow. pLDDT scores are shown in Additional file 3: Fig. S3

Here, the predicted structures of the native EnvC and the labeled EnvC-HA variants were compared by a MUSCLE sequence alignment (Fig. 6). As a computational prediction that has to be justified, it was shown that the integration of the HA-tag led to slight conformational changes while comparing the rank 001, model 3 prediction.

In consideration of the pLDDT score (< 50) minor structural changes of SP-HA and RPep-HA like the slight conformational displacement of the SP and the proline rich region (PRR) are not taken into account. Interestingly both constructs showed a small conversion of the TM (p15E) with a pLDDT score between 40 and 90 when compared to the wildtype (Fig. 6AI, AII, CI, CII). For the non-functional variant HA-VRA, structural prediction revealed an almost identical structure of the SP (AA 1–43) and TM (AA 473–623) when compared with PERV-C(5683). However, the introduction upstream of the VRA resulted in an intrinsically twisted loop formed by the HA-tag (AA 86–94) (pLDDT score < 50). In addition, the PRR (AA 247–285) underwent a major structural change (pLDDT score < 50). The PRR of PERV-C(5683) consists of a broad loop region, whereas the alteration of the HA-VRA PRR resulted in a twisted, thus compressed loop placed at a different angle (Fig. 6 B I, B II).

Alignment of the PERV-C-HA variants (Additional file 2: Fig. S2) showed that similar changes were observed for SP-HA and RPep-HA, whereas HA-VRA varies more from the others. While only minor differences were found in the TM (Additional file 2: Fig. S2 A), significant divergences were observed in the SP (Additional file 2: Fig. S2B) and PRR (Additional file 2: Fig. S2C).

### Infection assay of ST-IOWA/(PERV-C(5683) cells

**Fig. 7:**
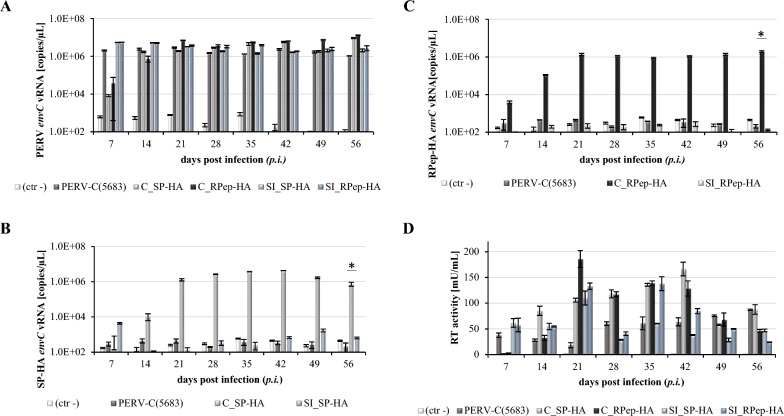
Quantification of vRNA and RT activity after challenging of ST-IOWA/PERV-C(5683) with PERV-C(5683)-HA viruses. Expression of PERV-C *env* vRNA was measured by qRT-PCR using **A** *envC* and **B**, **C** *envC-HA* specific primers. **D** Enzyme activity was monitored by RT assay. Native ST-IOWA cells were used as negative controls (ctr−), ST-IOWA cells infected with PERV-C(5683)-HA served as positive controls to confirm virus functionality. **A** Expression of *envC* vRNA was measured for all cells except for ST-IOWA (ctr−) starting from day 7 on. **B**, **C** *envC*-HA vRNA was detected for ST-IOWA cells infected with SP-HA or RPep-HA containing cell culture SNs but not for PERV-C(5683) positive cells challenged with PERV-C(5683)-HA. Significant means of < 0.05 according to Welch’s t test are indicated by (*). Analysis were performed in triplicates

**Fig. 8:**
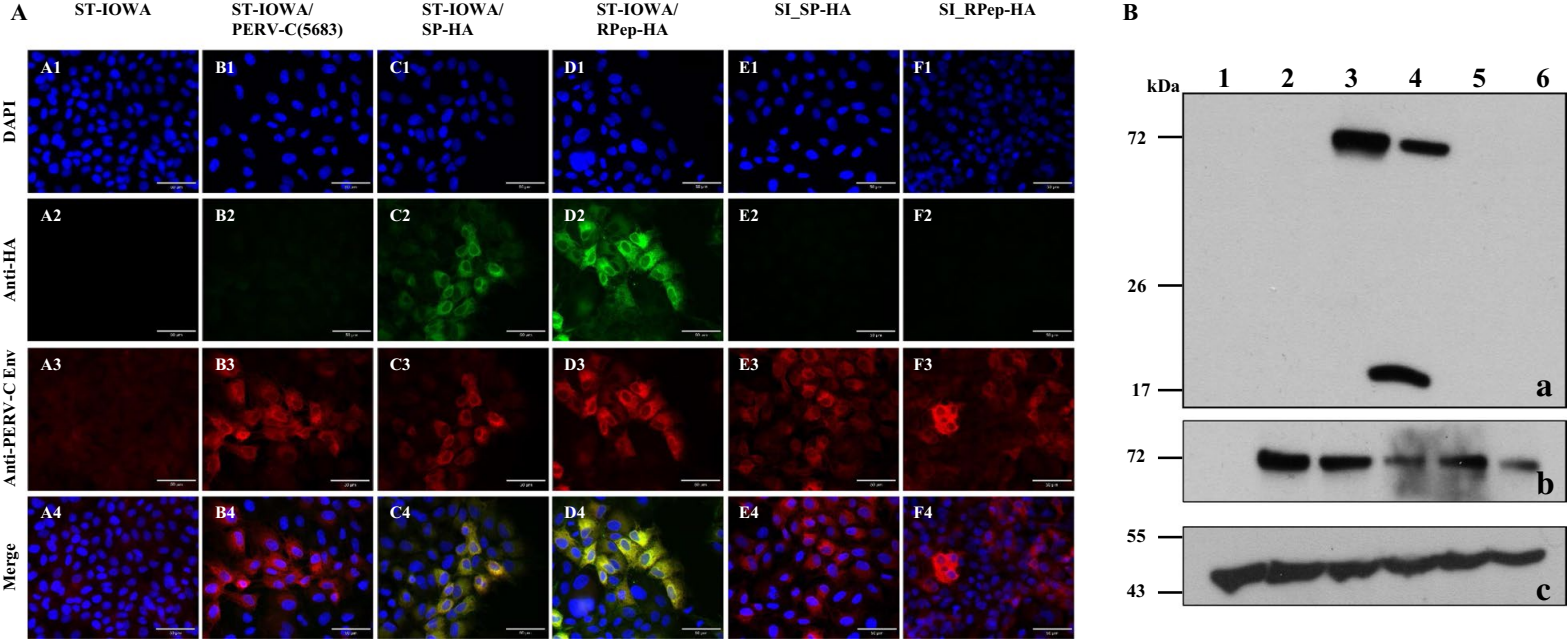
Immunofluorescence and WB analysis after superinfection. **A** Immunofluorescence of PERV proteins after superinfection of PERV-C(5683) positive ST-IOWA cells using antibodies directed against PERV-C Env and HA-tag. Protein expression was monitored at day 56 *p.i.*. A1-4: non-infected ST-IOWA (ctr−), B1-4: PERV-C(5683) positive ST-IOWA cells (ctr+) from Figure 5 D, B1-4 as a representative control, C1-4: ST-IOWA cells infected with SP-HA (ctr+) or D1-4: RPep-HA (ctr.+), E1-E4: ST-IOWA cells superinfected with SP-HA or F1-4: RPep-HA. PERV-C Env proteins were observed for all cells except for the (ctr−), while HA proteins only were present in primary infected control cells. **B** WB analysis of PERV-C proteins using antibodies directed against (a) the HA-tag or (b) PERV-C Env. WB was performed 56 days after superinfection of ST-IOWA-PERV-C(5683) cells with PERV-C(5683)-HA viruses. Native ST-IOWA cells were used as negative control (ctr−). ST-IOWA cells infected with virus present in SN that was also used for superinfection served as positive controls (ctr+). Lane 1: ST-IOWA (ctr−, lane 2: PERV-C(5683) positive ST-IOWA cells, lane 3–4: ST-IOWA cells infected with (3) SP-HA (ctr+) or (4) RPep-HA (ctr+), lane 5–6: PERV-C(5683) positive ST-IOWA cells superinfected with (5) SP-HA or (6) RPep-HA. **A** The EnvC-HA precursor (72.1 kDa) was detected for both SP-HA and RPep-HA. The corresponding cleaved product of SP-HA-SU is slightly below the precursor, thus indicated by a shadow. Since the HA-tag is located C-terminal in RPep-HA only the cleaved product TM-HA was detected. **B** For differentiation of EnvC and EnvC-HA, an anti-PERV-C Env(498) antiserum was used. PERV-C Env proteins were detected for all cells except for the negative control. **C** An anti‐beta‐actin WB was used as loading control

For the analysis of PERV-C SIR, virus producing ST-IOWA/PERV-C(5683) cells were challenged with PERV-C(5683)-HA viruses (SP-HA or RPep-HA) to set up superinfection (SI). The infection was followed for 56 days. Analysis was performed by qRT-PCR (Fig. 7A–C), RT activity assays (Fig. 7D), IF (Fig. 8A) as well as WB analysis (Fig. 8B). As a control for virus infectivity, i.e. functionality, native ST-IOWA cells were successfully infected with the same inoculum. For experimental readouts, PERV *envC* vRNA expression was quantified by qRT-PCR (Fig. 7A). Since SI cells and primary infected cells both express PERV-C *env* either with or without the HA-tag, levels of PERV-C *env* vRNA expression remained stable (10^6^–10^7^ copies/µL). Primary and secondary infections were distinguished by a subsequent qRT-PCR using *envC-HA* specific oligonucleotides (Table 3). The results show no expression in SI cells when compared to the controls. For primary infected ST-IOWA control cells *envC-HA* vRNA was 10^5^–10^6^ copies/µL and for secondary infected SI ST-IOWA/PERV-C(5683) cells it was 10^2^–10^3^ copies/µL (Fig. 7B, C). RT activity assays were used to monitor retroviral enzyme activity of the infected cells showing that all cells were RT positive (Fig. 7D). The presence of EnvC and EnvC-HA protein expression was analyzed by IF (Fig. 8A) and WB analysis (Fig. 8B) using a combination of PERV-C Env peptide and HA-specific antibodies. PERV-C Env proteins were detected in all cells except native ST-IOWA (ctr−) cells. HA proteins were only detected in the primary infected controls but not in the SI cells. Data confirmed that tagged viruses have the capacity to infect native ST-IOWA cells, however, they did not superinfect primary infected ST-IOWA/PERV-C(5683) cells.

**Table 3:** Oligonucleotide primers used for qRT-PCR

Primer	Target gene	Sequence 5′-3′	Accession No	Nucleotide pos	Amplicon
F	PERV *pol*	TCTCCCCAAGTAAAGCCTGAT	AJ133816.1	3119–3139	227 nt
R	PERV *pol*	ACTAGGATGCCCTGTTGGATTA	AJ133816.1	c3345–3324	
EnvC-for2	PERV *envC*	GTGCTCTCCTTCAGACCTAGATTAC	AM229312.2	9383–9407	184 nt
EnvC-R3_AWG	PERV *envC*	AGCCATTGGAGGCTCCAGCTGG	AM229312.2	c9566–9545	
RV_EnvC-HA	PERV *envC-HA*	CGCATAATCCGGCACATCATACGGATA			
EnvC-for3	PERV *envC-HA*	CGTTAAGCCGGCGCCACC	KY352351.2	6388–6405	146 nt
EnvC-for4	PERV *envC-HA*	GTTGGAGAAGCGTCGAAGGGAAAAGG	KY352351.2	8042–8067	277 nt

In addition, viral integration into the host genome of SI cells was excluded by PCR using PERV-C *env* and *envC-HA* specific primers (Table 3), analyzing gDNA of infected and SI cells (Additional file 4: Fig. S4). There was no HA-tagged proviral PERV-DNA detectable after superinfection.

## Discussion

Multiple proviral copies of PERV are spread throughout the pig genome. Some of them encode functional PERV of which only PERV-A, -B and -A/C are able to infect human cells in vitro. They are considered as a risk in the course of XTx. Up to now, there is no documented in vivo transmission of PERV except the effect of microchimerism after transplantation of porcine tissues and cells [50, 51]. However, the lack of evidence that polytropic PERV have no infectious potential for human xenotransplant recipients remains as a hurdle. Consequently, the generation of donor animals free of functional PERV and their protection from reinfection is a requirement for the development of porcine cell-based therapeutics. One approach was the genome-wide inactivation of PERV using CRISPR/Cas9 [34]. A beneficial side effect was that inactivated PK15 cells still producing inactive viral particles maintain cellular resistance to SI. This suggests a potential protective effect after inactivation of PERV RT and provided a first insight how to benefit from PERV SIR [36].

To carry on, we generated HA-tagged PERV-C(5683) as a model virus to inoculate PERV-C(5683) positive ST-IOWA cells in vitro, and confirmed that SIR is also valid for PERV-C. To this end, PERV-C(5683) viruses were labeled with an HA sequence tag located at three different regions of *envC* (SP-HA, HA-VRA and RPep-HA). SP-HA and RPep-HA viruses showed infectivity comparable to PERV-C(5683). In contrast, the integration of an HA-tag N-terminal to the VRA region resulted in an impaired viral infectivity. This finding indicates the structural importance of this region that was impeded e.g. by disrupting a binding motive or interfering with the protein structure while integrating new amino acids. Evidence that structural changes in HA-VRA lead to a loss of recombination capacity has been strengthened by computational analysis using AlphaFold2. The major changes are located in a loop region with limited prediction accuracy (pLDDT score < 50). Nevertheless, it is worth mentioning as they differentiate the nonfunctional variant HA-VRA from PERV-C(5683) and the functional variants SP-HA and RPep-HA.

The responsiveness to insertion mutagenesis marks the VRA upstream region as a potential target for further virus inactivation studies, and might be applicable to other PERV classes.

The PERV HA-tagging allows a differentiation of primary and secondary infection as shown for PERV-C positive ST-IOWA cells. Thus, PERV-C SIR was confirmed. In addition to this study, HA-tagged PERV-C provides a useful tool to further study host–pathogen interactions, in vitro and in vivo. The positions that have been proven suitable for tag introduction in PERV-C are applicable for other PERV classes e.g. PERV-A, -B, -A/C.

In terms of SI, the aspect that ST-IOWA/PERV-C(5683) cells challenged by the same type of virus remain unaffected, raises questions regarding the mechanism. Either the presence of viral proteins, virus particle formation or the release of functional viruses itself seems to prevent the host-cell from new infection by an identical or similar virus. For PERV-C, it was proven that an integration of the tagged virus into the host genome did not occur. Even if SI cells were negative for *envC-HA* DNA, the entry of viral RNA into the host cell cannot be excluded. The inability to integrate could be caused by inadequate cell entry e.g. attachment, receptor binding or fusion or due to an insufficient post-entry affecting the uncoating or the integration itself. Data from MuLV and FV studies [41, 45, 52] suggest that retroviral SI is blocked at an early stage, e.g. by masking or downregulating the receptor through Env-binding or by a general upregulation of antiviral factors. For PERV, an analogous mechanism is expected. In PK15 and ST-IOWA cells the porcine PERV-A receptor (POPAR) is expressed on the RNA level and ST-IOWA cells show a basal expression of PERV-A *env* RNA [36]. PERV-RNA expression is not sufficient to restrict SI, which indicates an inhibition on the protein level.

Consequently, the usage of PERV proteins as a protection against exogenous PERV infection is obvious. Takeuchi et al., 1998, demonstrated in their host range and interference studies that cells that actively produce e.g. PERV-A or -B are not susceptible to pseudotype virus of the same class [6]. Similar results were shown by Argaw et al. [53]. Infection with PERV excludes reinfection with PERV of the same class. Infection with different PERV classes is possible due to different host receptors. Since the tropism of PERV is determined by Env protein, it is the most likely factor to mediate SIR. Even if non-Env-mediated SIR mechanisms are described e.g. Gag/MuLV [54, 55] it seems unlikely for PERV. Gag-mediated SIR would be expected to protect against all PERV classes as it is highly conserved. Its mode of action could be that of a cofactor or carrier protein.

Eventually, SIR prevents amplification of PERV in the host genome through reinfection. On the other hand, ERVs including PERVs can amplify through intracellular retrotransposition [56, 57], by transcription and subsequent reverse transcription leading to ERV sequences that are missing distinct parts of the LTR. Last but not least, in silico screening showed that *env* gene loss is associated with a significant (up to 30-fold) increase in genome copy number, implying that such *env*-less ERVs can be considered genomic superspreaders [58]. This could be an explanation for multiple dysfunctional PERV copies in the pig genomes.

The newly generated PERV-C(5683)-HA viruses are a suitable tool for gaining knowledge on PERV-C host–pathogen interaction. An application of already existing cellular defense mechanisms against PERV infection on the basis of SIR is a novel approach that will contribute to increase the virus safety of xenotransplants.

## Conclusion

Elimination of functional PERV in XTx must be ensured. In recent years, great success in this field has been achieved through the selection of PERV-C-free pigs and the genome-wide inactivation of PERV using CRISPR/Cas9. However, preventing reinfection, i.e. SI, of donor animals with PERV is of importance. Therefore, we have developed HA-tagged functional PERV confirming that the mechanisms of SIR also applies to PERV-C. It opens up a new possibility using a natural existing protective mechanism against PERV, thereby supporting virus safety of XTx.

## Methods

### Design and preparation of PERV-C-HA plasmids

Design of HA-tagged PERV-C was performed starting with the viral backbone of PERV-C(5683) (KY352351.2) [26] cloned in pBluescript SK (+) (Agilent Technologies, Germany). *EnvC* of PERV-C(5683) was structurally analyzed using the open source tool PSIPRED [59] to highlight sections of amino acids that do not bear structural motifs (Additional file 1: Fig. S1A). Only sequence sections defined as coil were selected for integration of the tag. Integration into helixes or ß-strands was avoided. Based on sequence alignments, structural analysis and on published functional studies [11, 26, 53, 60] the HA-tag was introduced at AA 44–52, AA 86–94 or AA 639–647 resulting in SP-HA, HA-VRA and RPep-HA, respectively. Corresponding sequences are shown in Additional file 1: S1B–D.

Integration of the HA-tag into PERV-C(5683) was performed by site directed mutagenesis using the Q5^®^ Site-Directed Mutagenesis Kit (New England Biolabs, Germany) following the manufacturer’s instruction. Primers used are shown in Table 1.

### Cell lines and viruses

PERV‐C was harvested from producer cell line ST-IOWA, carrying the molecular clone PERV-C(5683), KY352351.2. Native ST-IOWA cells [9, 61] were used as negative control. These cells are susceptible to PERV-C infection and are RT negative as they are free of functional PERV-A and -B [62]. Cells were cultured as described previously [26, 63]. Cells and corresponding RT activities of virus‐containing SNs used for infection experiments are listed in Table 2.

### In vitro* transfection of ST-IOWA with PERV-C*

For in vitro transfection, PERV-C DNA was purified by the EndoFree Plasmid Maxi Kit (Qiagen, Germany) as recommended by the manufacturer. ST-IOWA cells were seeded in triplicates of 2 × 10^5^ ST-IOWA cells/well in six-well culture plates (Sarstedt, Germany) 24 h prior transfection. Transfection was performed using the FuGene^®^ HD transfection reagent (Promega, USA) according to the manufacturer's instructions with a ratio of transfection reagent to DNA of 3:1.

### In vitro* infection of ST-IOWA and ST-IOWA/PERV-C(5683)*

For in vitro infection, triplicates of 2 × 10^5^ ST-IOWA cells/well, were seeded in six-well culture plates (Sarstedt, Germany) 24 h prior infection. Cells were incubated with 1 mL/well cell-free SN containing replication-competent PERV-C in different amounts according to RT activity values (Table 2) for 24 h. After washing the cells three times with 2 mL/well phosphate buffered saline (PBS) to remove input virus, cells were further cultivated for 56 days. Quantification of PERV-C expression and RT activity took place weekly from cell‐free SN of pooled triplicates. Cell‐free SN aliquots were stored at – 80 °C until testing.

### Preparation of viral RNA and quantification of viral RNA expression

Isolation of viral RNA from cell-free SNs was performed using the QIAamp^®^ Viral RNA Mini Kit (Qiagen, Germany), according to manufacturer’s instruction. One-step qRT-PCR was performed on PERV *pol*, *env-C* and *env-C*-HA in a LightCycler^®^ 480 (Roche Life Science, Swiss) using the QuantiTect SYBR Green RT-PCR Kit (Qiagen, Germany) as recommended by the manufacturer. Detection and quantification of PERV-C *env* and *pol* vRNA was performed as described before, using primer pair EnvC-for2/EnvC-R3_AWG or F/R [36, 63]. For the quantification of *env-C*-HA, cycling conditions were as follows: RT‐reaction (50 °C, 30 min); denaturation (95 °C, 15 min); 40 cycles of amplification (94 °C, 15 s, denaturation; 68 °C, 30 s, annealing; and 72 °C, 30 s, elongation). Melting curve analysis was performed as follows: 95 °C, 10 s, 65 °C, 10 s, slowly heating up to 97 °C, continuously measuring the fluorescence and cooling (40 °C, 30 s). Based on the tag position two different forward primers were used for *envC-HA* quantification. The reverse primer binds specifically in the HA-tag and thus can be used for SP-HA and RPep-HA (Table 3). Primer binding sites of the forward primers were selected so that amplicons suitable for qRT PCR are formed.

### Reverse transcriptase activity test

The activity of viral RT in cell free SNs of PERV producer and infected cells was measured using the in-house HS-MN RT kit (Cavidi Ab, Sweden) in accordance to the manufacturer’s instructions of protocol B. Samples were tested in triplicates (50 µL each) and mean value and standard deviation were determined. The standard dilution series covered RT activity values from 0.11 mU/mL to 33.97 mU/mL. RT activity values below this were considered to be RT negative and defined the limit of detection.

### Western blot

Cell lysates from transfected ST-IOWA cells were analyzed for the presence of structural proteins by WB. Briefly, samples were subjected to SDS‐PAGE (10%) and transferred onto Immobilon‐P PVDF membranes (Merck Millipore, Germany). For functional analyses of HA-tagged PERV-C(5683) viruses, membrane was blocked with 0.1% TBS‐T containing 5% (w/v) skimmed milk (AppliChem, Germany) and proteins were detected with polyclonal rabbit Anti-PERV-C(498) antiserum (1:250) (peptide sequence: H2N-TGQRPPTQGPGPSSNI-COOH, manufactured by Eurogentec, Belgium) in 0.1% TBS‐T containing 5% (w/v) skimmed milk (AppliChem, Germany) at room temperature for 1 h. Infection assays of ST-IOWA/(PERV-C(5683) cells were analyzed for presence of Env and HA using the polyclonal rabbit anti-PERV-C(498) antiserum (1:250) and a mouse Anti-HA antibody (1 µg/mL) (GenScript, USA) in 0.1% TBS‐T containing 5% (w/v) skimmed milk (AppliChem, Germany) at room temperature for 1 h. Peroxidase-conjugated donkey anti-rabbit (Dianova, Germany); 1:15,000) or goat anti-mouse (Dianova, Germany); 1:10,000) secondary antibodies were incubated for 1 h in 0.1% TBS-T at room temperature. Anti‐beta‐actin (Abcam, USA); 1:10,000) was used as control. For detection, an Amersham Hyperfilm^™^ and ECL Western Blotting Detection Reagents (GE Healthcare, USA) were used.

### Indirect immunofluorescence microscopy

Detection of PERV particles by IF microscopy was performed as described previously [64, 65]. Briefly, semi-confluent to confluent cells were seeded on glass coverslips and fixed with 2% formaldehyde (4% ROTI^®^Histofix (Carl Roth, Germany) in PBS) in PBS for 30–45 min. Coverslips were stored at 4–8 °C until use. Permeabilization took place by incubation with 0.5% Triton X-100 (Sigma-Aldrich, USA) for 10 min. Cells were washed three times with PBS, blocked with 1% BSA in PBS for 10 min and incubated with PERV-C(498) antiserum for 30 min. Cy3-conjugated anti-rabbit IgG (Dianova, Germany) was used for detection. FITC-conjugated anti-HA antibody was added for incubation with Cy5-conjugated anti rabbit IgG (Dianova, Germany). Samples were embedded in Fluoroshield^™^ mounting medium containing 4′, 6-Diamidino-2-phenylindole dihydrochloride (DAPI) (Sigma-Aldrich, USA) and images were acquired using the Revolve 4 microscope (ECHO, USA).

### Electron microscopy

EM of PERV-C-HA cells was performed according to standard procedures as described previously [21]. Briefly, cells were fixed with 2.5% glutaraldehyde (Sigma‐Aldrich, USA) in culture medium for 45 min at room temperature. Cells were washed two times with PBS. Subsequently, sample preparation was performed in the electron microscopy facility at Paul-Ehrlich Institut. Cells were scraped off the culture flask and gently mixed with 2% warm liquid agarose. After cooling and gelling, small agarose blocks containing the cells were cut and post‐fixed with 2% osmium tetroxide (Sigma‐Aldrich, USA) in PBS and treated with 1% tannic acid (Carl Roth, Germany). Cells were gradually dehydrated in a series of ethanol baths and embedded in Epoxy resin (Sigma‐Aldrich, USA). Polymerization lasted for 48 h at 60 °C, afterwards ultrathin sections were cut and stained with 2% uranyl acetate (Merck, Germany) for 15 min followed by 2% lead citrate (Serva, Germany) for 5 min. Sections were examined in a Jeol JEM 1400 Flash electron microscope using electron filtering (ESI) mode.

### Integration control of PERV-C by PCR

Proviral PERV-C-HA was analyzed by PCR on purified gDNA from cells after superinfection. Target cell gDNA was purified using the QIAamp^®^ DNA Blood Mini Kit (Qiagen, Germany) as recommended by the manufacturer. PCR was performed with 100 ng gDNA with primer pairs EnvC for3 and rvEnvC-HA for PERV-C(5683)-SP-HA and EnvC for4 and rvEnvC-HA for PERV-C(5683)-RPep-HA. An EnvC specific PCR using the primers EnvC for3 and EnvC-R3_AWG was performed as control. gDNA of ST-IOWA cells served as negative control. Q5 DNA Polymerase (NEB, Germany) was used following manufacturers’ instructions.

### Structural prediction using AlphaFold2

Structural prediction of PERV-C(5683) Env as well as generated HA-tagged EnvCs was performed using AlphaFold2 via the ColabFold pipeline applying mostly default parameters [49, 66]. AlphaFold2 provides a computational approach that has been used to predict many protein structures with almost experimental accuracy [49]. Resulting structure models gaining the highest model confidence for each protein were analyzed for structural changes using Chimera [67]. pLDDT scores obtained after the AlphaFold prediction are shown in Additional file 3: Fig. S3.

### Statistical analysis

Statistical significance of RNA samples was determined by Welch’s t-test with 0.05 as the threshold.

## Supplementary Information

**Additional file 1: Figure S1.** AA-sequence and secondary structure analysis of PERV-C(5683) (KY352351.2) (PSIPRED [47]). **A** PSIPRED analysis taken as basis for the HA-tag introduction. Black arrows indicate positions of HA-tag integration. Env specific domains like SP, variable regions and PRR are framed in black. **B–D** EnvC AA-sequences of **B** SP-HA, **C** HA-VRA and **D** RPep-HA. Position and sequence of the HA-tag is marked in blue. EnvC-SP, -variable regions and -PRR are underlined, in bold.

**Additional file 2: Figure S2.** Structural comparison of PERV-C(5683)-HA variants. Sequence motifs are labeled: SP (green), VRA (light blue), VRB (dark blue), PRR (purple) and RPep (yellow). **A** MUSCLE Alignment of SP-HA, HA-VRA and RPep-HA, whole Env proteins. Comparison of the HA-tagged PERVs revealed a high similarity of SP-HA and RPep-HA. Structural differences of HA-VRA, SP-HA and RPep-HA are most noticeable in the SP (**B**) and PRR (**C**). Only minor differences were found in the TM. Here, the SP of HA-VRA stays close in the for PERV-C(5683) predicted position (see Fig. 6), while the SP of SP-HA and RPep-HA undergoes a structural alteration. The PRR of HA-VRA shows a more twisted and a compressed loop compared to SP-HA and RPep-HA. pLDDT scores are shown in Figure S3.

**Additional file 3: Figure S3.** pLDDT scores obtained after AlphaFold prediction. Scores for all predicted models (rank 1–5) of PERV-C(5683) as well as for PERV-C(5683)-HA viruses are shown. Residues with pLDDT ≥ 90 have very high model confidence, residues with 90 > pLDDT ≥ 70 are classified as confident. Scores with 70 > pLDDT ≥ 50 have low confidence, and residues with pLDDT < 50 correspond to very low confidence [68].

**Additional file 4: Figure S4.** Control of proviral integration of PERV-C(5683)-HA after superinfection. Genomic DNA of superinfected cells (56 days *p.i.*) was used for *envC* (**A**) and *envC-HA* (**B**) specific PCRs. Lane 1: ST-IOWA (ctr−), lane 2: PERV-C(5683) positive ST-IOWA cells, lane 3: ST-IOWA infected with SP-HA (ctr1+), lane 4: ST-IOWA positive for PERV-C(5683) superinfected with SP-HA, lane 5: ST-IOWA infected with RPep-HA (ctr2+), lane 6: ST-IOWA positive for PERV-C(5683) superinfected with RPep-HA. The HA-tag was detectable in ST-IOWA cells infected with SP-HA or RPep-HA but not in superinfected cells indicating that superinfection related integration does not occur.

## Data Availability

All data generated or analyzed in this study are included in this published article including the supplementary information.

## Abbreviations

AA: Amino acid
BHK-21: Syrian hamster fibroblast cell line
DAPI: 4′, 6-Diamidino-2-phenylindole dihydrochloride
DPF: Designated pathogen free
*env*: Envelope gene
Env: Envelope protein
EM: Electron microscopy
FV: Foamy virus
Fv4: Retrovirus-related Env polyprotein from the *Fv4* locus
gDNA: Genomic DNA
HA: Hemagglutinin
HIV: Human immunodeficiency virus
IF: Immunofluorescence
kDa: KiloDalton
LTR: Long terminal repeat
MEM: Minimum essential medium
MuLV: Murine leukemia virus
NEB: New England BioLabs
PBS: Phosphate buffered saline
PCMV: Porcine cytomegalovirus
PERV: Porcine endogenous retrovirus
*p.i.*: Post infection
PK15 cells: Porcine kidney cells
POPAR: Porcine PERV-A receptor
PRR: Proline rich region
*p.t.*: Post transfection
qRT-PCR: Quantitative one-step reverse transcriptase polymerase chain reaction
RT: Reverse transcriptase
RPep: R-peptide
SI: Superinfected
SIR: Superinfection resistance
SP: Signal peptide
SN: Supernatant
ST-IOWA: Swine testis cell line
SU: Surface unit
TM: Transmembrane unit
VRA: Variable region A
vRNA: Viral RNA
WB: Western blot
XTx: Xenotransplantation
