# Correction: Analysis of PERV-C superinfection resistance using HA-tagged viruses

**DOI:** 10.1186/s12977-025-00659-0

**Published:** 2025-02-27

**Authors:** Merle Flecks, Nicole Fischer, Jacomina Krijnse Locker, Ralf R. Tönjes, Antonia W. Godehardt

**Affiliations:** 1 Division of Haematology, Cell and Gene Therapy, Paul-Ehrlich-Institut, Langen, Germany; 2 Loewe‑DRUID Research Group, Electron Microscopy of Pathogens, Paul-Ehrlich-Institut, Langen, Germany

**Correction: Retrovirology (2023) 20:14** 10.1186/s12977-023-00630-x

Following publication of the original article [1], it was pointed out that Fig. 8B was accidentally uploaded a second time as Fig. 3B during the submission process. The correct Fig. 3B has now been swapped in.

Additionally, the legend of Fig. 8A for subpanels B1–B4 has been amended for clarity to read “…B1–4: PERV-C(5683) positive ST-IOWA cells (ctr+) from Fig. 5 D, B1–4 as a representative control…”

The original article has been corrected.
