# Supplementary material for: Analysis of PERV-C superinfection resistance using HA-tagged viruses
Source: Retrovirology. 2023 Aug 21;20:14. doi: 10.1186/s12977-023-00630-x (PMC10440901; doi:10.1186/s12977-023-00630-x)
Supplement: Supplementary file 1 — Additional file 1: Figure S1. AA-sequence and secondary structure analysis of PERV-C(5683) (KY352351.2) (PSIPRED [47]). A PSIPRED analysis taken as basis for the HA-tag introduction. Black arrows indicate positions of HA-tag integration. Env specific domains like SP, variable regions and PRR are framed in black. B–D EnvC AA-sequences of B SP-HA, C HA-VRA and D RPep-HA. Position and sequence of the HA-tag is marked in blue. EnvC-SP, -variable regions and -PRR are underlined, in bold. [file 12977_2023_630_MOESM1_ESM.pptx]

## Slide 1
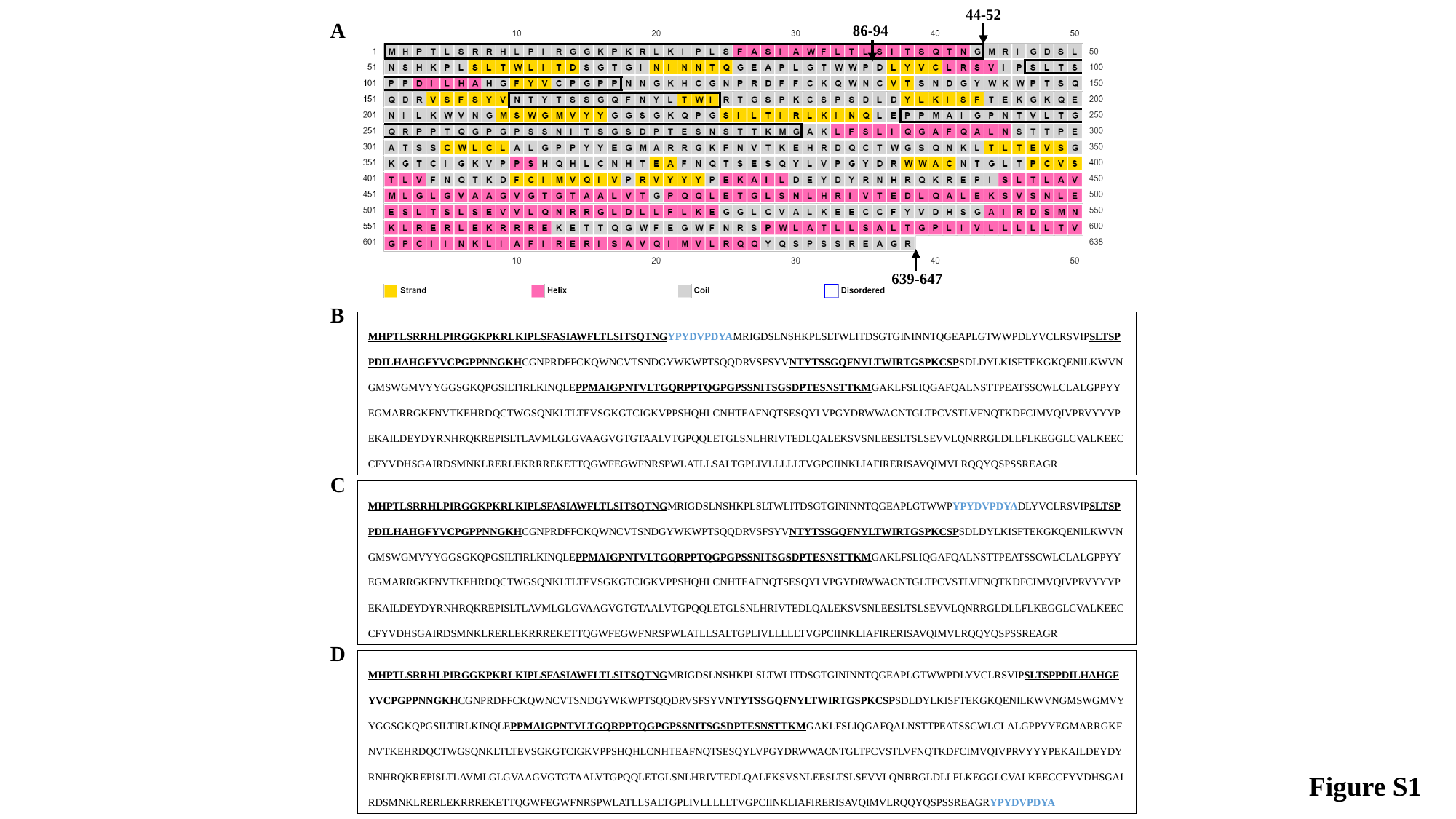

44-52
A
86-94
639-647
B
MHPTLSRRHLPIRGGKPKRLKIPLSFASIAWFLTLSITSQTNGYPYDVPDYAMRIGDSLNSHKPLSLTWLITDSGTGININNTQGEAPLGTWWPDLYVCLRSVIPSLTSPPDILHAHGFYVCPGPPNNGKHCGNPRDFFCKQWNCVTSNDGYWKWPTSQQDRVSFSYVNTYTSSGQFNYLTWIRTGSPKCSPSDLDYLKISFTEKGKQENILKWVNGMSWGMVYYGGSGKQPGSILTIRLKINQLEPPMAIGPNTVLTGQRPPTQGPGPSSNITSGSDPTESNSTTKMGAKLFSLIQGAFQALNSTTPEATSSCWLCLALGPPYYEGMARRGKFNVTKEHRDQCTWGSQNKLTLTEVSGKGTCIGKVPPSHQHLCNHTEAFNQTSESQYLVPGYDRWWACNTGLTPCVSTLVFNQTKDFCIMVQIVPRVYYYPEKAILDEYDYRNHRQKREPISLTLAVMLGLGVAAGVGTGTAALVTGPQQLETGLSNLHRIVTEDLQALEKSVSNLEESLTSLSEVVLQNRRGLDLLFLKEGGLCVALKEECCFYVDHSGAIRDSMNKLRERLEKRRREKETTQGWFEGWFNRSPWLATLLSALTGPLIVLLLLLTVGPCIINKLIAFIRERISAVQIMVLRQQYQSPSSREAGR
C
MHPTLSRRHLPIRGGKPKRLKIPLSFASIAWFLTLSITSQTNGMRIGDSLNSHKPLSLTWLITDSGTGININNTQGEAPLGTWWPYPYDVPDYADLYVCLRSVIPSLTSPPDILHAHGFYVCPGPPNNGKHCGNPRDFFCKQWNCVTSNDGYWKWPTSQQDRVSFSYVNTYTSSGQFNYLTWIRTGSPKCSPSDLDYLKISFTEKGKQENILKWVNGMSWGMVYYGGSGKQPGSILTIRLKINQLEPPMAIGPNTVLTGQRPPTQGPGPSSNITSGSDPTESNSTTKMGAKLFSLIQGAFQALNSTTPEATSSCWLCLALGPPYYEGMARRGKFNVTKEHRDQCTWGSQNKLTLTEVSGKGTCIGKVPPSHQHLCNHTEAFNQTSESQYLVPGYDRWWACNTGLTPCVSTLVFNQTKDFCIMVQIVPRVYYYPEKAILDEYDYRNHRQKREPISLTLAVMLGLGVAAGVGTGTAALVTGPQQLETGLSNLHRIVTEDLQALEKSVSNLEESLTSLSEVVLQNRRGLDLLFLKEGGLCVALKEECCFYVDHSGAIRDSMNKLRERLEKRRREKETTQGWFEGWFNRSPWLATLLSALTGPLIVLLLLLTVGPCIINKLIAFIRERISAVQIMVLRQQYQSPSSREAGR
D
MHPTLSRRHLPIRGGKPKRLKIPLSFASIAWFLTLSITSQTNGMRIGDSLNSHKPLSLTWLITDSGTGININNTQGEAPLGTWWPDLYVCLRSVIPSLTSPPDILHAHGFYVCPGPPNNGKHCGNPRDFFCKQWNCVTSNDGYWKWPTSQQDRVSFSYVNTYTSSGQFNYLTWIRTGSPKCSPSDLDYLKISFTEKGKQENILKWVNGMSWGMVYYGGSGKQPGSILTIRLKINQLEPPMAIGPNTVLTGQRPPTQGPGPSSNITSGSDPTESNSTTKMGAKLFSLIQGAFQALNSTTPEATSSCWLCLALGPPYYEGMARRGKFNVTKEHRDQCTWGSQNKLTLTEVSGKGTCIGKVPPSHQHLCNHTEAFNQTSESQYLVPGYDRWWACNTGLTPCVSTLVFNQTKDFCIMVQIVPRVYYYPEKAILDEYDYRNHRQKREPISLTLAVMLGLGVAAGVGTGTAALVTGPQQLETGLSNLHRIVTEDLQALEKSVSNLEESLTSLSEVVLQNRRGLDLLFLKEGGLCVALKEECCFYVDHSGAIRDSMNKLRERLEKRRREKETTQGWFEGWFNRSPWLATLLSALTGPLIVLLLLLTVGPCIINKLIAFIRERISAVQIMVLRQQYQSPSSREAGRYPYDVPDYA
Figure S1
