# Supplementary figures and images for: Analysis of PERV-C superinfection resistance using HA-tagged viruses
Source: Retrovirology. 2023 Aug 21;20:14. doi: 10.1186/s12977-023-00630-x (PMC10440901; doi:10.1186/s12977-023-00630-x)

## Slide 1
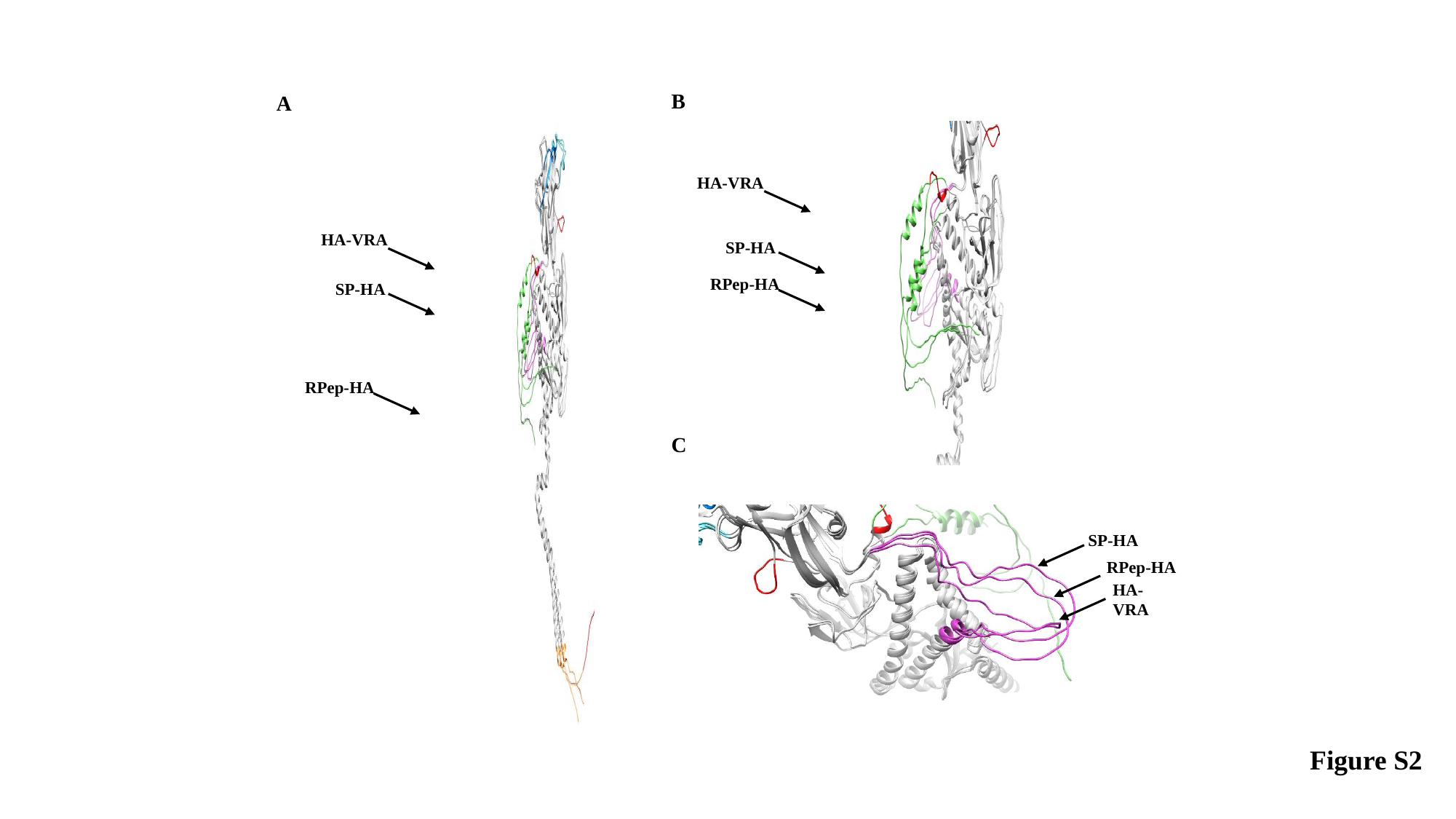

B
A
HA-VRA
HA-VRA
SP-HA
RPep-HA
SP-HA
RPep-HA
C
SP-HA
RPep-HA
HA-VRA
Figure S2

Supplement: Supplementary file 2 — Additional file 2: Figure S2. Structural comparison of PERV-C(5683)-HA variants. Sequence motifs are labeled: SP (green), VRA (light blue), VRB (dark blue), PRR (purple) and RPep (yellow). A MUSCLE Alignment of SP-HA, HA-VRA and RPep-HA, whole Env proteins. Comparison of the HA-tagged PERVs revealed a high similarity of SP-HA and RPep-HA. Structural differences of HA-VRA, SP-HA and RPep-HA are most noticeable in the SP (B) and PRR (C). Only minor differences were found in the TM. Here, the SP of HA-VRA stays close in the for PERV-C(5683) predicted position (see Fig. 6), while the SP of SP-HA and RPep-HA undergoes a structural alteration. The PRR of HA-VRA shows a more twisted and a compressed loop compared to SP-HA and RPep-HA. pLDDT scores are shown in Figure S3. [file 12977_2023_630_MOESM2_ESM.pptx]

## Slide 1
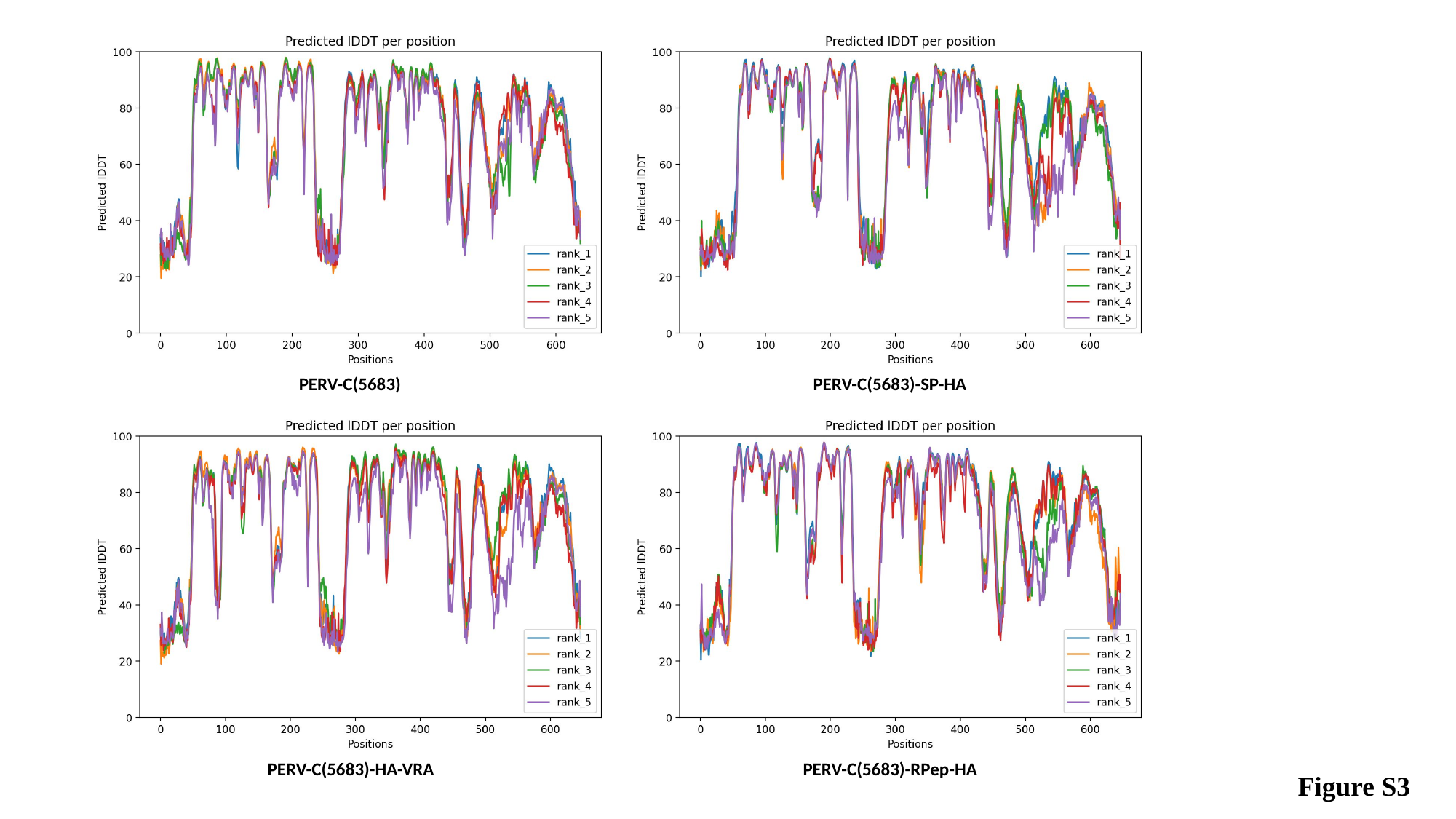

PERV-C(5683)
PERV-C(5683)-SP-HA
PERV-C(5683)-HA-VRA
PERV-C(5683)-RPep-HA
Figure S3

Supplement: Supplementary file 3 — Additional file 3: Figure S3. pLDDT scores obtained after AlphaFold prediction. Scores for all predicted models (rank 1–5) of PERV-C(5683) as well as for PERV-C(5683)-HA viruses are shown. Residues with pLDDT ≥ 90 have very high model confidence, residues with 90 > pLDDT ≥ 70 are classified as confident. Scores with 70 > pLDDT ≥ 50 have low confidence, and residues with pLDDT < 50 correspond to very low confidence [68]. [file 12977_2023_630_MOESM3_ESM.pptx]

## Slide 1
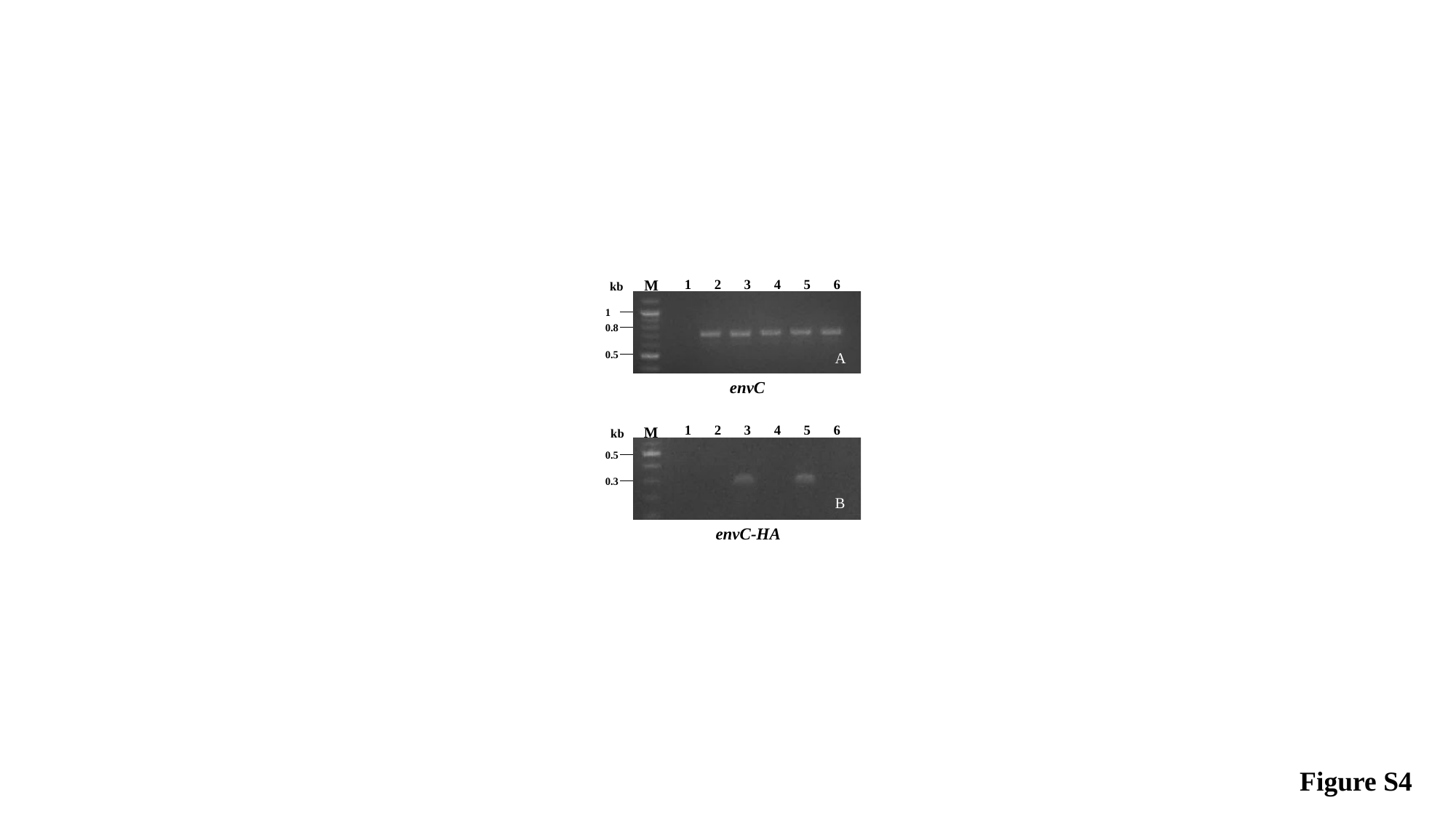

M
1
2
3
4
5
6
kb
1
0.8
0.5
A
envC
1
2
3
4
5
6
M
kb
0.5
0.3
B
envC-HA
Figure S4

Supplement: Supplementary file 4 — Additional file 4: Figure S4. Control of proviral integration of PERV-C(5683)-HA after superinfection. Genomic DNA of superinfected cells (56 days p.i.) was used for envC (A) and envC-HA (B) specific PCRs. Lane 1: ST-IOWA (ctr−), lane 2: PERV-C(5683) positive ST-IOWA cells, lane 3: ST-IOWA infected with SP-HA (ctr1+), lane 4: ST-IOWA positive for PERV-C(5683) superinfected with SP-HA, lane 5: ST-IOWA infected with RPep-HA (ctr2+), lane 6: ST-IOWA positive for PERV-C(5683) superinfected with RPep-HA. The HA-tag was detectable in ST-IOWA cells infected with SP-HA or RPep-HA but not in superinfected cells indicating that superinfection related integration does not occur. [file 12977_2023_630_MOESM4_ESM.pptx]
